# Impacts of Aerobic Exercise on Depression-Like Behaviors in Chronic Unpredictable Mild Stress Mice and Related Factors in the AMPK/PGC-1α Pathway

**DOI:** 10.3390/ijerph17062042

**Published:** 2020-03-19

**Authors:** Jia Luo, Changfa Tang, Xiaobin Chen, Zhanbing Ren, Honglin Qu, Rong Chen, Zhen Tong

**Affiliations:** 1 Key Laboratory of Kinesiology Evaluation and Recovery of General Administration of Sport of China, Sports Science institute of Hunan, Changsha 410012, China; luojia0301@163.com; 2 Department of Physical Education, Hunan Normal University, Changsha 410012, China; Tangchangfa@sina.com (C.T.); chenrong1206@163.com (R.C.); TZMYTH@163.com (Z.T.); 3 Department of Sports and Health, Guangzhou Sport University, Guangzhou 510500, China; 15975533891@139.com; 4 Department of Physical Education, Shenzhen University, Shenzhen 518060, China; 5 Department of Physical Education, Yichun College, Yichun 336000, China; quhonglin20040125@126.com

**Keywords:** depression, chronic stress, AMPK/PGC-1α, exercise

## Abstract

This study was to study the impact of aerobic exercises on the chronic unpredictable mild stress (CUMS) in mice, and to discuss the possible mechanism from the skeletal muscle AMPK/PGC-1α energy metabolism signaling pathway. The healthy male mice were randomly divided into Control Group (CG), Model Group (MG), and Model Exercise Group (ME).Twelve stress methods were adopted for four weeks (28 days) to establish the depression model. ME was subject to aerobic training plan after the model was established. The weight of the mice was recorded weekly. After the experimental intervention, the three groups of mice were subjected to behavioral assessment tests. Western blotting, RT-PCR, and ELISA were performed to test AMPK, p-AMPK, PGC-1α, and ATP in skeletal muscle. There were no significant difference in body weight between the three groups. CUMS leaded to significant decline in behavioral scores. and the p-AMPK and PGC-1α decreased significantly. But boosted ATP content. Aerobic exercise enhanced the expressions of p-AMPK and PGC-1α, increased the ratio of p-AMPK/AMPK, boosted ATP content. And improved behavioral scores significantly. Chronic stress-induced depression-like behavior was improved significantly by Aerobic exercise. The mechanism of aerobic exercise for improving depressive symptoms in mice with chronic stress depression may be related to influence AMPK/PGC-1α pathway.

## 1. Introduction

The impact of moderate physical activities on chronic diseases has been a hot topic in the field of exercise physiology and chronic diseases. The physical exercise has become an acknowledged, safe, affordable, and important means of promoting health. Aerobic exercises, in particular, have been shown to have a beneficial effect on delaying aging, inhibiting apoptosis, managing hyperlipidemia, improving brain cognition, and treating dementia [1,2,3,4], which has been a boon for improving the life quality of human. 

Depression is a psychogenic disease characterized by a persistently gloomy mood. Today, globally, about 350 million patients are suffering from depression. Among all the lethal and disabling diseases in the world, depression ranks the ninth, next to diseases such as heart disease and stroke [5]. The pathogenesis of depression is extremely complicated. Although there have been many pieces of research on depression in recent years, it has not been fully explained. Therefore, further research on the pathogenesis and treatment of depression is of great importance. Most researchers believe that the cause of depression is the changes in the brain nervous system, such as low monoamines such as norepinephrine and 5-hydroxy tryptamine(5-HT), and endocrine axis disorders [6]. However, the impact of depression on the peripheral should not be ignored. According to clinical reports of depression patients, physical symptoms such as muscle-ache and fatigue are common in depression [7]. Recently, scholars have focused on the connecting mechanism of the peripheral and central nervous systems, which provides new research directions for depression. The peripheral mechanism may provide new perspectives for the treatment of depression. 

Studies have shown that aerobic exercise can significantly reduce the symptoms of depression and is an “effective medicine” to treat depression. The mechanism is to improve intravascular growth factors to extend capillaries, to stimulate neuron regeneration, and to regulate mood and cognition [8]; to enhance and regulate neurotrophin such as brain-derived neurotrophic facto (BDNF) and improve the plasticity of the brain [9]. Exercise can reduce the level of peroxidation, prevent mitochondrial dysfunction, and reduce brain damage [10]. The energy metabolism disturbance has recently been speculated to be an important factor in mediating depression. The evidence is that clinical studies have found that for depression patients, the glucose metabolism significantly slows in their brains and the ATP level is reduced in their skeletal muscle tissues [11]. The low ATP level may result in physical and emotional symptoms which are closely related to depression [12]. As an important organ for energy metabolism in the human body, the skeletal muscle is sensitive to energy metabolism and serves as the main organ that maintains body movements. The current research on the impact of aerobic exercise on depression still focuses on its effect on the central nervous system, while its impact on the skeletal muscle energy metabolism remains undiscovered. 

Adenosine 5′-monophosphate (AMP)-activated protein kinase (AMPK) is a core factor to maintain energy homeostasis, and its main function is closely related to ATP level [13]. Due to stress, the ATP level falls, AMP/ATP ratio increases, and AMPK is activated [14]. peroxisome proliferator-activated receptorγcoactivator-1 (PGC-1α) is a downstream factor of AMPK and its transcriptional activity can be enhanced by AMPK [15]. AMPK/PGC-1α is important for balancing the body energy [16]. Therefore, by establishing a chronic stress depression model in mice, this study explores the changes in ATP level, AMPK, and PGC-1α related factors, and discusses the possible mechanism of the rehabilitative effect of aerobic exercise on depression from the perspective of skeletal muscle energy metabolism. 

## 2. Methods

### 2.1. Subjects and Groups

The subjects are 45 male eight-week-old KM mice. The lab mice and food were purchased from Hunan SJA Laboratory Animal and the certificate number is SCXK (xiang) 2016-0002. Treatment of experimental animals complies with the Guide for the Care and Use of Laboratory Animals from the National Research Council. The mice lived a regular life for 1 week after purchase and were randomly divided into Control Group (CG), Model Group (MG), and Model Exercise Group (ME). Each group had 15 mice who were divided into two cages, with seven or eight mice per cage. The bedding was changed regularly every week. The mice were provided with enough water during the whole period. The humidity was kept at 40–60%, and the temperature at 20–25%. Adaptive feed lasted for three days. CG was fed conventionally and may eat and drink freely. MG and ME were constantly simulated for 28 days by stress factors to establish the depression model in mice and may eat and drink freely. The weight of the mice was recorded weekly. 

### 2.2. Modeling Depression in Mice

The establishment of models of depression in mice referred to the chronic stress stimulation model adopted by Willner [17] et al. and Xin Xin [18] et al. Chronic unpredictable mild stress (CUMS) was performed on MG and ME, including restraint, lightly squeezing the tail, wet bedding, horizontal shaking, swimming in cold water (4 °C), swimming in high temperature (38 °C), no water, no food, slanting cage, white noise, constant lighting, and day-night reversal. One or two types of simulation were performed daily. The same type of simulation was not performed continuously. The stress regime lasted for four weeks (28 days). 

### 2.3. Behavioral Assessment 

Behavioral assessment tests include tail suspension test and forced swim test.

Tail suspension test: The lower third of the tail was fixed onto an iron rack. The mouse was suspended with its head facing downward. A stopwatch was used to measure time. The mouse was hung for six minutes. The immobile duration during the last four minutes of hanging was recorded. 

Forced swim test: A 30cm-tall 15cm-wide container was used. 20cm water was added into the container. The temperature was about 25 °C. The mouse was put into the container and it was guaranteed that its tail cannot reach the bottom. A stopwatch was used to measure time. The mouse remained in the water for six minutes. The total immobile duration during the last four minutes was recorded. 

### 2.4. Aerobic Exercise Plan

Referring to the exercise plan by Bedford [19] and exercise plan adopted by the lab before, the treadmill running was optimized. After successful modelling, exercise intervention started on ME. In the first three days, the slope of the treadmill was set to 0. The mice run at 8 m per minute for 30 min per time. On the fourth day, the speed was increased to 10 m per minute and the slope remained the same. After that, the mice performed treadmill running once per day, 60 min per time, six times per week for six weeks.

### 2.5. Sample Preparation

The mice in CG, MG, and ME were anesthetized 24 h after the end of the last behavioral test to prepare the sample. The dead mice were fixed on the operation table, the fur was cleaned with 75% ethanol, and the quadriceps femoris was taken. The muscle tissue was put into cold physiological saline and washed clean. The adipose tissue and connective tissue were removed from the muscle. The excessive physiological saline was absorbed by filter paper. The prepared muscle tissue was cut horizontally into two parts and transferred into liquid nitrogen for instant freezing. Later, the tissue was transferred into a −80 °C ultra-low temperature freezer.

### 2.6. Diagnostic Assay

Skeletal muscle ATP level was measured by Elisa. Western Blot was used to detect skeletal muscle AMPK, p-AMPK, and PGC-1α protein expression. Realtime-PCR was used to detect skeletal muscle AMPK and PGC-1α mRNA expression.

#### 2.6.1. Western Blot

Take 0.025 g tissue, add 200 ulripa to crack and grind, crack on ice for 10 min. Centrifugation. Follow the instructions of the BCA protein quantitative Kit (wellbio), Determination of protein concentration. After electrophoresis, the proteins were transferred electrophoretically onto a nitrocellulose membrane as described previously, After blocking with 5% nonfat dried milk powder/TBS/0.1% Tween (1.5 h at room temperature), membranes were probed with the Primary antibodies overnight at 4 °C. antibody include PGC-1α (66369-1-Ig, Mouse, 1:2000, proteintech of America); AMPK (66536-1-Ig; Mouse; 1:2000; proteintech of America); P-AMPK (ab168346; Rabbit; 1:2000; abcam of England) and GAPDH(10494-1-AP; Rabbit; 1:3000; proteintech of America). Secondary antibody was incubated for 90min, Dilute with 1 × TBST, HRP goat anti-mouse IgG (1:5000, proteintech of America), HRP goat anti-rabbit IgG (1:6000, proteintech of America), using the enhanced chemiluminescence technique (ECL; Pierce Chemical Co., Rockford, IL, USA). All assays were performed at least three times. 

#### 2.6.2. Realtime-PCR

Extraction of total RNA by conventional Trizol reagent, follow the instructions of the SYBR Premix Ex TaqTMII (Takara) to reverse transcription of c D-NA, RT-PCR reaction after reverse transcription, by usie apparatus:ABI 7900HT, The reaction conditions are 95 °C 10 min, 1cycle pre-denaturation; 95 °C 15 s, 60 °C 30 s, 65 °C 30 s, 40-cycle PCR reaction; 72 °C for 10 min. Repeat three wells per sample, Calculation of relative gene expression by 2^−△△Ct^ method. The base sequence is reported in Table 1.

#### 2.6.3. Elisa

Test kit purchased from Sanway Biotechnology Co., Ltd. in hunan. Use the Multiscan Spectrum (product: MB-530), Measuring the absorbance of each hole in order of 450 nm wavelength (optical density), and it should be performed within 15 min after adding the stop solution.

### 2.7. Data Processing

SPSS19.0 was used for statistical analysis and graph production. Data are expressed as mean ± standard deviation (X ± S). Comparisons between groups were performed by one-way anova; and multiple comparisons were performed by Tukey test anova. *p* < 0.05 indicates the difference is statistically significant and *p* < 0.01 indicates the difference is highly significant. 

## 3. Results

### 3.1. Weight Results

The weight change of mice in each group is shown in Table 2. After all the exercise, MG < ME< CG in terms of the total weight. Compared with CG, the weights of MG and ME were no significant difference (*p* > 0.05). The MG compared with ME was no significant difference either (*p* > 0.05). The Weight is reported in Table 2.

### 3.2. Behavioral Results

**Figure 1:**
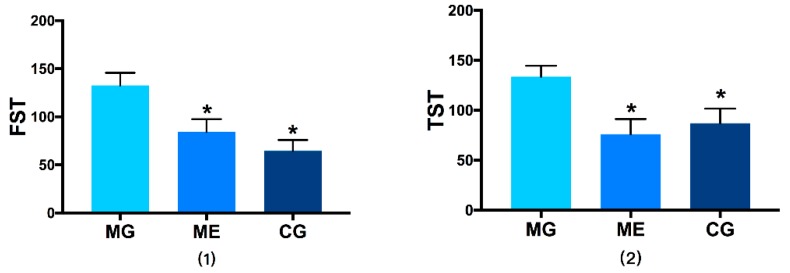
(1) shows the results of the forced swim test, Picture (2) shows the results of the tail suspension test. Control Group = CG, Model Group = MG, and Model Exercise Group = ME. * indicates significant difference compared with MG (*p* < 0.01).

In forced swim test and tail suspension test, compared with CG, the immobile duration of MG was significantly longer (*p* < 0.01), indicating that the d esperate behavior was reinforced and their instinct for survival was low, which means the chronic stress depression model was well-established. Aerobic exercise significantly reduced the immobility duration of ME (*p* < 0.01). The results are reported in Figure 1.

### 3.3. Western Blot Results

#### 3.3.1. Changes in AMPK/p-AMPK Protein Expression

**Figure 2:**
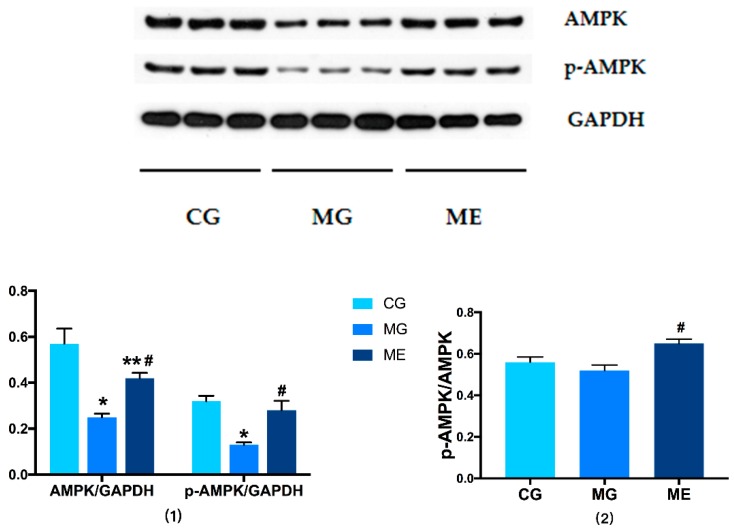
Protein: AMPK, Internal reference protein: GAPDH. (1) shows the results of AMPK/GAPDH and p-AMPK/GAPDH, (2) shows the results of p-AMPK/AMPK. Control Group = CG, Model Group = MG, and Model Exercise Group = ME. * indicates significant difference compared with CG (*p* < 0.01); ** indicates significant difference compared with CG (*p* < 0.05); ^#^ indicates significant difference compared with MG (*p* < 0.01).

In terms of AMPK phosphorylation level, there was significant difference between MG and CG. The *p*-AMPK in MG was significantly downregulated (*p* < 0.01), ME was significantly upregulated compared with MG (*p* < 0.01), and there was no significant difference between ME and CG. The ratio of pAMPK/AMPK in ME was significantly upregulated than MG (*p* < 0.01). There were no significant difference between MG and CG. The results are reported in Figure 2.

#### 3.3.2. Changes in PGC-1α Protein Expression 

**Figure 3:**
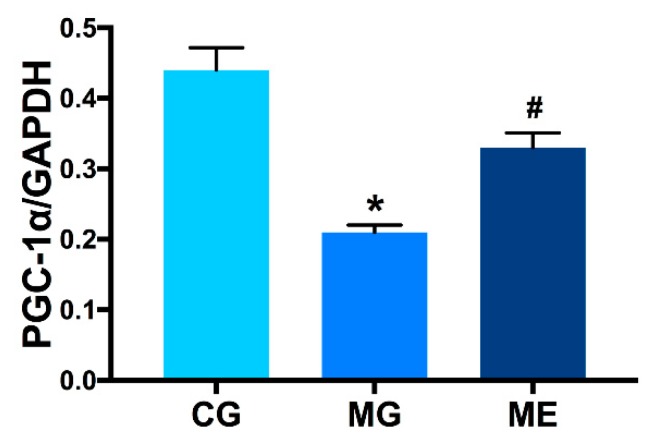
Protein: PGC-1α, Internal reference protein: GAPDH. Control Group = CG, Model Group = MG, and Model Exercise Group = ME; * indicates *p* < 0.01 compared with CG; ^#^ indicates *p* < 0.01 compared to MG.

In terms of the expression of PGC-1α in skeletal muscle, CG > ME > MG. There was a highly significant difference between MG and CG (*p* < 0.01). There was a highly significant difference between ME and MG (*p* < 0.01). ME compared with CG was no significant difference (*p* > 0.05).The results are reported in Figure 3.

### 3.4. RT-PCR on AMPK and PGC-1αmRNA Expression

#### AMPK and PGC-1αmRNA Expression

**Figure 4:**
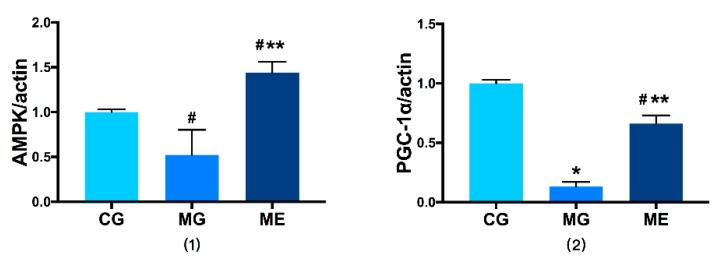
Gene: PGC-1α, Internal reference gene: actin. (1) shows the results of AMPK/action, (2) shows the results of PGC-1α/action. Control Group = CG, Model Group = MG, and Model Exercise Group = ME; * indicates *p* < 0.01 compared with CG; ^#^ indicates *p* < 0.05 compared with CG, ** indicates *p* < 0.01 compared with MG.

In terms of the level of gene transcription, in AMPKαmRNA expression, MG was downregulated compared to CG (*p* < 0.05), and ME was upregulated compared to MG. Both were significantly different (*p* < 0.01). Compared with CG, MG saw highly significant trend of downregulation in PGC-1αmRNA expression (*p* < 0.01). In ME, the expression level of PGC-1αmRNA was downregulated compared with MG (*p* < 0.01), lower than CG (*p* < 0.05). The results are reported in Figure 4.

### 3.5. Elisa Results

**Figure 5:**
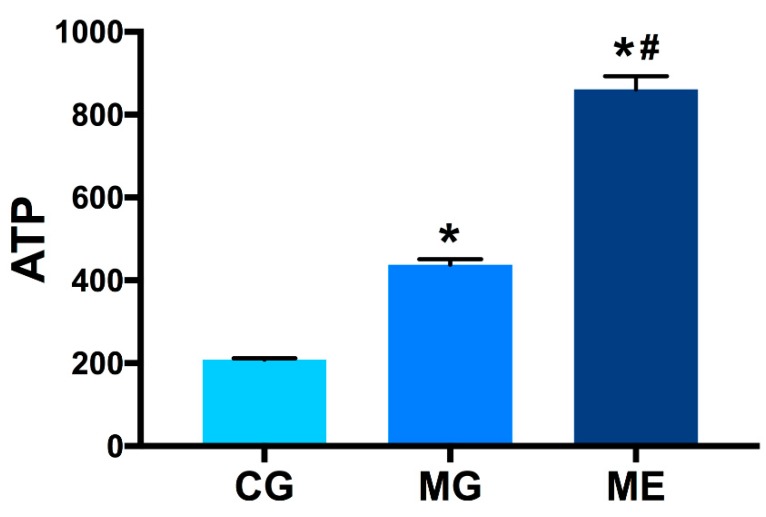
Content in skeletal muscle tissue homogenate. Control Group = CG, Model Group = MG, and Model Exercise Group = ME.* indicates *p* < 0.01 compared with CG; ^#^ indicates *p* < 0.01 compared with MG.

In terms of ATP level, ME > MG > CG. Compared with CG, the ATP level of MG was significantly higher (*p* < 0.01), and the ATP level in ME was more than twice as high as that in MG (*p* < 0.01). The results are reported in Figure 5.

## 4. Discussion 

### 4.1. Discussion on Chronic Stress-Induced Depression Model in Mice 

Depression is a neurological dysfunction whose core symptoms are persistently low mood, lack of pleasure, irritability, decreased attention, and abnormal appetite, and sleeping disorder. Besides the high suicide rate and high recurrence rate, depression is also closely related to coronary arterial diseases and type II diabetes [20]. In the past few decades, animal models of depression have been of great importance for the study of the pathogenesis of depression. Studies have suggested that the onset of depression is related to stress in life. The chronic stress is sudden, compulsive, and long-term. A large number of studies have shown that animal models of depression induced by chronic stress are more stable and reliable [21,22,23]. The methods used to establish the stress model vary in different studies. So far, besides chronic unpredictable mild stress modeling, widely used stress-induced modeling for rodents have included chronic restraint stress modeling, learned helplessness modeling, social deprivation modeling, etc. Chronic unpredictable mild stress (CUMS) is a depression modeling method that simulates human’s living environment. It features diverse, random, and unstable stimulus and is similar to situations in human life to a large extent. The earliest CUMS was seen in the experiment by Katz [24] et al. in 1983, where stimulus including electrical stimuli was adopted on rats as an intervention for 21 days to establish the model. The results showed that the scores for open field test was reduced and the ability to respond to the stimulus was significantly reduced. Twelve types of stimulation were used in this study. One or two types of stimulation were selected each day for 28 days. It was found out that the weigh increase of the mice slowed down but there was no significant difference. The behavioral score was significantly reduced (*p* < 0.01). The animal model was successfully established. Wan Renling [25] et al. selected six-week-old mice and nine types of stimulation, and one type of stimulation was adopted every day. After 21 days, the weight loss of mice was significant (*p* < 0.01) and the behavioral score was significantly reduced (*p* < 0.05). Huang Qiaoling [26] selected five-week-old mice and seven types of stress factors, and one type of stress factor was performed every day. After 21 days, the weight gain slowed down but there was no significant difference, and the behavioral score reduced significantly (*p* < 0.05). Zhong et al. [27] selected eight- to ten-week-old mice and eleven stress factors, and two or three types of stimulation were performed every day. After a constant stimulation of two to three weeks, there was significant difference in the weight and the behavioral scores of the mice (*p* < 0.01). Jiang Ning [28] et al. selected more than ten types of stimulus and used two or three types every day. After five weeks, the behavioral score of the mice was significantly reduced (*p* < 0.01). Therefore, different intensities of stimulation result in different depression-like symptoms in mice. The more stimulus adopted, the more significant the effect; also, age seems to have an impact on the response to stress. 

### 4.2. Impacts of Aerobic Exercise on the Behavior and Weight of Mice with CUMS-Induced Depression-Like Behaviors

Studies have suggested that the criteria for evaluating the effectiveness of animal models are: (1) the symptoms are similar to those of human diseases; (2) behavioral changes can be objectively monitored; and (3) the behavioral change can be reversed by effective anti-depression treatment. Behavioral changes are commonly seen in depression patients, such as dislike of movement, appetite loss, weight loss, lack of interest in external things, and learning ability decline. The behavioral testing methods of animal models currently include sucrose preference test, open field test, forced swim test, tail suspension test, and water maze test. A large number of reports have suggested that the symptoms are similar to those of human depression and the methods are highly operable. Sucrose preference test can be applied to determine the anhedonia in the model; tail suspension test and forced swimming test can be used to determine the reduced desire to move, reduced curiosity, and reinforced desperate behaviors in the model. Therefore, behavioral evaluation methods in animal study have become classic methods for the discussion on models of depression. Research has suggested that weight loss is one of the nine major physical signs of depression [29], and therefore is also selected as one of the indicators for model assessment.

The results of this study suggest that 28 days of CUMS resulted in a significant reduction in behavioral scores and weight gain in MG and ME, but there was no statistical significance. After introducing aerobic exercise intervention, the behavioral score was significantly enhanced and the weight gain was slightly improved. The results of this study mostly agree with the results of forced swimming tests and tail suspension tests in domestic and foreign studies on the rehabilitative impact of aerobic exercise on CUMS-induced depression. Chronic stress results in the significant decline in the mobility of the model, while aerobic exercise can reverse this phenomenon [30]. However, the result of weight change disagrees to some of the previous studies. Wen et al. [31] have suggested that CUS significantly reduced the weight, while the intervention of aerobic exercise can reduce the degree of weight loss. Zhuang et al. [32] found that chronic stress led to a significant reduction in the weight and sucrose consumption. Aerobic exercise led to increased sucrose consumption, but caused slow weight gain. It is inferred that it may be because of stress intensity and exercise plan. Besides, Liu et al. [33] studied the differences in depression-like behaviors of modelled mice of different genders and found that gender seemed to have an impact on chronic stress modeling. Female mice underwent more significant changes in behaviors and weight than male mice. Meanwhile, it is reported that the incidence of females is two to three times higher than that of males [34]. While so far, most studies have chosen male mice, and few have chosen female mice. Overall, CUMS has a high success rate to establish models of depression and is an ideal method for depression modelling. However, when designing the experiment, researchers should select appropriate stress methods and intensity, and the possibility of the adaptability of animal models to the stress should be considered. In addition, when assessing the model, it is more reliable and comprehensive to select multiple indicators and methods. 

### 4.3. Impacts of Aerobic Exercise on AMPK/PGC-1α of Mice with CUMS-Induced Depression-Like Behaviors

The energy for various physical activities comes from the ATP produced by the catabolism of glucose and fat. When the ATP level decreases, a self-regulatory mechanism is started in the cell to reduce energy consumption, prevent resource exhaustion, and maintain physiological functions [35]. AMPK is a protein kinase activated by AMP. Through the changes in ATP, ADP or ATP/AMP, activated phosphorylation regulates the regulation and decomposition of energy [36,37,38]. AMPK has three subunits, namely α, β and γ. The CBS domain on the γ-subunit can combine ATP, which enables AMPK to respond to changes in the ATP-AMP ratio [36]. When intracellular ATP level changes, activated AMPK further plays a regulatory role [39]. PGC-1α is peroxisome proliferator-activated receptor gamma coactivator 1-alpha and AMPK is the key inducer of PGC-1α signaling, constituting two key regulatory factors in the AMPK /PGC-1 energy metabolism signal axis [40]. Many studies were about the correlation between AMPK/PGC-1 and obesity, diabetes, and metabolic syndrome [39,41]. Recently, scholars have suggested that energy metabolism disorders are very likely to be an important pathological mechanism of depression. The evidence is that a significant decrease in the mitochondrial ATP level was found in the brain and muscle tissues of depression patients [42,43,44,45]. However, the specific mechanism is still unknown. 

The results showed that the AMPK and the expressions of *p*-AMPK protein and mRNA of MG was downregulated compared with CG. The expressions of PGC-1α protein and mRNA were both lower than CG. This agrees to the results of studies by Fang et al. [46], Odaira et al. [47], and Agudelo et al. [48], which indicates that the AMPK /PGC-1 signaling pathway factor is activated by chronic stress and that AMPK energy metabolism pathway disorders happen in the skeletal muscles of depression patients. In contrast to the results of this experimental study, Cao et al. [49] found that mice with chronic social failure had lower levels of ATP in the brain. Gardner et al. [45] proposed a reduction in skeletal muscle ATP in patients with clinical depression. In this experiment, an increase in ATP content was observed in the skeletal muscle of the model group. Consider whether the chronic stress-induced depression model will have different results due to the stress response, and it is interesting that some studies have found that stress leads to increased excitability of neural networks, stress activates the GR receptor on neurons, which leads to increased extracellular levels of glutamate. This glutamate may subsequently activate NMDA receptors on astrocytes, and these then produce ATP [50].This seems to point out the direction for the difference results, futhermore, usually a lower AMPK activation is correlated with a lower ATP production, but this study show there is a lower AMPK activation in the MG group, but a higher ATP levels. Although AMPK is regulated by ATP and AMP/ATP, but it is still unclear how exactly ATP and AMP are regulated, and whether there are other intermediates, and then In addition to ATP and AMP, AMPK is involved in complex mechanisms of regulation. For example, AMPK’s upstream kinases liver kinase B1 (LKB1), TGF-βactivated kinase TAK1, calmodulin-dependent protein kinase CaMK, etc. can all regulate AMPK. Although the distribution of these factors in various tissues of the body are different, it is not known whether they play a role in the skeletal muscle of mice caused by chronic stress. Yuan [51] find that chronic stress activates SGK1 and suppresses the expression of LKB1 via inhibitory phosphorylation of FOXO3a. Downregulated LKB1 contributes to reduced activation of AMPK, but reports are rare on the periphery mechanism of depression, especially in skeletal muscle, and the reasons need to be further observed.

We found that aerobic exercise up regulate the expression of protein significantly in the ratio of p-AMPK/AMPK and mRNA in MG, and also significantly upregulate the PGC-1α protein and mRNA. The expression of ATP level in skeletal muscle rose significantly, indicating that aerobic exercise may relieve depression by activating the AMPK energy metabolism pathway in the skeletal muscle. In recent years, the impact of aerobic exercise on the expression of AMPK and PGC-1α has been frequently reported by researchers such as Koltai et al. [52], Wang Dalei et al. [53], and Wang Yangjie et al. [54]. The results of this study agree with their research. Aerobic exercise has a significant impact on the AMPK phosphorylation level, and upregulate the expression of PGC-1α and gene. Study results of increasing ATP levels through administration to effectively improve depression-like behavior in depressed mice [49]. It is suggested that the mechanism of aerobic exercise to relieve depression may be related to the ATP level that affects skeletal muscle and thus regulate the AMPK energy metabolism pathway.

## 5. Conclusions

Chronic stress-induced depression-like behavior was improved significantly by Aerobic exercise. The mechanism of aerobic exercise for improving depressive symptoms in mice with chronic stress depression may be related to influence AMPK/PGC-1α pathway.

## Figures and Tables

**Table 1:** RT-PCR base sequence.

Base Sequence	
**GAPDH**	F ACAGCAACAGGGTGGTGGAC R TTTGAGGGTGCAGCGAACTT
PGC-1α	F TGATGTGAATGACTTGGATACAGACA R GCTCATTGTTGTACTGGTTGGATATG
AMPK	F AAACCCACAGAAATCCAAACAC R CCTTCCATTCATAGTCCAACTG

Primer design by Sangon Biotech in Shanghai, forward = F, reverse = R.

**Table 2:** Weight of mice.

Group	Before Experiment	After Modelling	After Exercise Intervention
CG	41.68 ± 1.3503	53.47 ± 2.8909	61.37 ± 2.5877
MG	41.54 ± 1.4773	49.86 ± 1.4607	55.56 ± 1.2855
ME	42.13 ± 1.2811	49.16 ± 1.0023	58.4 ± 1.5269

Control Group = CG, Model Group = MG, and Model Exercise Group = ME. (X ± S, *n* = 15, Unit: g).
